# Comparison of Prognosis between Minimally Invasive and Abdominal Radical Hysterectomy for Patients with Early-Stage Cervical Cancer

**DOI:** 10.3390/curroncol29040185

**Published:** 2022-03-24

**Authors:** Tomohito Tanaka, Shoko Ueda, Shunsuke Miyamoto, Sousuke Hashida, Shinichi Terada, Hiromi Konishi, Yuhei Kogata, Kohei Taniguchi, Kazumasa Komura, Masahide Ohmichi

**Affiliations:** 1 Department of Obstetrics and Gynecology, Educational Foundation of Osaka Medical and Pharmaceutical University, Takatsuki 569-8686, Japan; shouko.ueda@ompu.ac.jp (S.U.); shunsuke.miyamoto@ompu.ac.jp (S.M.); sosuke.hashida@ompu.ac.jp (S.H.); shinichi.terada@ompu.ac.jp (S.T.); hiromi.konishi@ompu.ac.jp (H.K.); yuhei.kogata@ompu.ac.jp (Y.K.); m-ohmichi@ompu.ac.jp (M.O.); 2 Translational Research Program, Educational Foundation of Osaka Medical and Pharmaceutical University, Takatsuki 569-8686, Japan; kohei.taniguchi@ompu.ac.jp (K.T.); kazumasa.komura@ompu.ac.jp (K.K.)

**Keywords:** uterine cervical cancer, radical hysterectomy, minimally invasive surgery

## Abstract

Minimally invasive surgery (MIS) is performed to treat cervical cancer patients; however, a recent study showed that MIS was associated with higher recurrence and death rate compared with abdominal radical hysterectomy (ARH). In the current study, the prognosis of patients with early-stage cervical cancer who underwent MIS with vaginal closure or ARH was evaluated. One hundred and eighty-two patients underwent radical hysterectomy for cervical cancer with stage of IA2, IB1, and IIA1. MIS was performed by laparoscopy or a robot using the vaginal closure method. Disease-free survival (DFS) and overall survival (OS) were evaluated between the groups. Among the patients, 67 underwent MIS and 115 underwent ARH. The recurrence rate was 4.5% in MIS patients and 3.5% in ARH patients with a median follow-up (interquartile range) of 36 (18–60) and 78 (48–102) months, respectively. DFS and OS were not different between the groups (3y-DFS, 95.3% vs. 96.1%, *p* = 0.6; 3y-OS, 100% vs. 100%, *p* = 0.06). In early-stage cervical cancer patients, MIS with vaginal closure did not increase the risk for recurrence or death. Surgical techniques and procedures to avoid spillage of tumor cells could be important for a better prognosis.

## 1. Introduction

Although abdominal radical hysterectomy (ARH) has been performed on cervical cancer patients, the popularity of minimally invasive surgery (MIS), including laparoscopic radical hysterectomy and robotic radical hysterectomy, has been increasing among these patients in the last decade [1]. However, the first large randomized prospective study that compared MIS and ARH, the LACC trial (LACC ClinicalTrials.gov number, NCT00614211), demonstrated that MIS was associated with lower rates of disease-free survival (DFS) and overall survival (OS) [2]. Several factors may explain the differences in prognosis between minimally invasive and open approaches in the results of that study. These factors include spillage or implantation of tumor cells using a manipulator, the effect of the insufflation gas on tumor cell growth or spread, and the experience of surgeons; however, the reason for the difference is unknown [2]. We evaluated the prognosis of early-stage cervical cancer patients who underwent MIS with vaginal closure or ARH.

## 2. Materials and Methods

### 2.1. Study Particitants

Among the early-stage cervical cancer patients who underwent laparoscopic, robotic, or abdominal radical hysterectomy at the Educational Foundation of Osaka Medical and Pharmaceutical University in Japan between January 2013 and January 2021, 182 were enrolled in the study. Laparoscopic and robotic radical hysterectomy was performed using the vaginal closure method. All methods were performed in accordance with relevant guidelines and regulations. The study protocol was approved by the ethical guidelines of the 1975 Declaration of Helsinki, as revised in 1983, and was approved by the Educational Foundation of Osaka Medical and Pharmaceutical University Clinical Research Review Board (IRB protocol ID: 2020-087 and 2013-053). Informed consent was obtained in the form of an opt-out on the website. Patients who rejected the opt-out were excluded.

All cases were classified using pathology findings based on the Federation of Gynecology and Obstetrics (FIGO) Grading system in 2009. Patients who met the following criteria were eligible for inclusion in the study: (1) those who underwent laparoscopic, robotic, or abdominal radical hysterectomy (type III with nerve sparing) for cervical cancer; (2) patients whose medical records contained accurate information; (3) patients who did not undergo chemotherapy or radiotherapy before surgery; and (4) those whose stage was IA2, IB1, or IIA1.

### 2.2. Surgical Procedure

**Figure 1:**
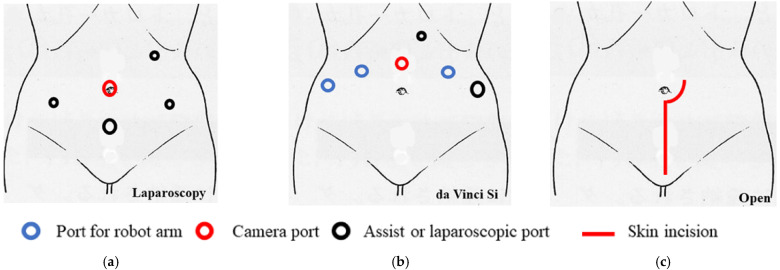
The port replacement and skin incision. (**a**) Total laparoscopic radical hysterectomy was performed as a standard five-port technique without intrauterine manipulation in the lithotomy–Trendelenburg position. (**b**) Robotic radical hysterectomy was performed using the da Vinci Si system in the lithotomy–Trendelenburg position. Four robot ports, one 5-mm trocar, and one 12-mm trocar were placed. (**c**) Abdominal radical hysterectomy was performed with vertical skin incision.

We previously reported the surgical procedure [3] and the procedure for the sentinel lymph node biopsy [4,5]. Total laparoscopic radical hysterectomy (type III) with vaginal closure was performed as a standard five-port technique without intrauterine manipulation in the lithotomy–Trendelenburg position (Figure 1a). For surgery, vessel sealing devices, including LigaSure (Medtronic, Minneapolis, MN, USA), Enseal (Ethicon; Johnson & Johnson, Cincinnati, OH, USA), and Thunderbeat (Olympus Medical Systems Corp., Tokyo, Japan), were used.

Robotic radical hysterectomy with vaginal closure was performed using the da Vinci Si system (Intuitive Surgical, Sunnyvale, CA, USA) in the lithotomy–Trendelenburg position. Four robot ports, one 5-mm trocar, and one 12-mm trocar were placed (Figure 1b). A uterine manipulator was not used. The procedures were performed in the same way as the laparoscopic procedures described above.

Abdominal radical hysterectomy was performed with a vertical skin incision (Figure 1c). The procedures were the same as those for laparoscopy; however, vaginal closure was not performed.

### 2.3. Approach to Vaginal Closure

**Figure 2:**
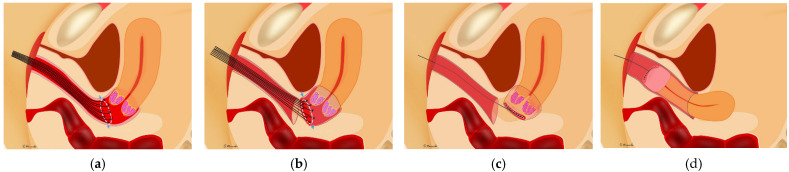
Vaginal closure for minimally invasive radical hysterectomy for cervical cancer. (**a**) Several sutures were placed on the cut line of vagina transvaginally. (**b**) The vaginal mucosa was cut in a circle 3 mm outside the knots with pulling of the sutures. (**c**) The vaginal cuff of the uterine side was closed with running sutures; the cervical cancer was completely covered with vaginal mucosa. (**d**) After the circumferential colpotomy was performed under laparoscopy or using a robot, the uterus was removed transvaginally.

Vaginal closure was performed after all uterine ligaments were cut; this meant that the uterus was connected only by the vagina. The procedure was performed transvaginally without active insufflation. Several knots of 1-0 silk were placed on the cut line of the vagina (Figure 2a). The vaginal mucosa was cut 3 mm outside the knot distally (Figure 2b). The running sutures that were placed on the vaginal cuff of the uterine side were tightened; the cervical cancer was covered with the vaginal mucosa to avoid spillage of cancer cells (Figure 2c). Then, circumferential colpotomy was performed with a laparoscope or robot under active insufflation using monopolar scissors. After removal of the uterus, the vaginal cuff was closed using a laparoscope or robot (Figure 2d) [3].

### 2.4. Statistic Analysis

All statistical analyses were performed using the JMP software package (version 15.1.1) (SAS Institute Japan, Tokyo, Japan). Continuous variables are expressed as median and interquartile range or mean ± standard deviation. The Mann–Whitney U-test was used to compare continuous variables, and Fisher’s exact test was used to compare frequencies. Fisher’s exact test with Bonferroni’s correction was used to compare frequencies among the three groups. Survival was estimated using the Kaplan–Meier method with log-rank test. A propensity score-matching analysis was performed to ensure that both groups were homogenous and comparable. *p*-values < 0.05 were considered to indicate statistical significance.

## 3. Results

**Figure 3:**
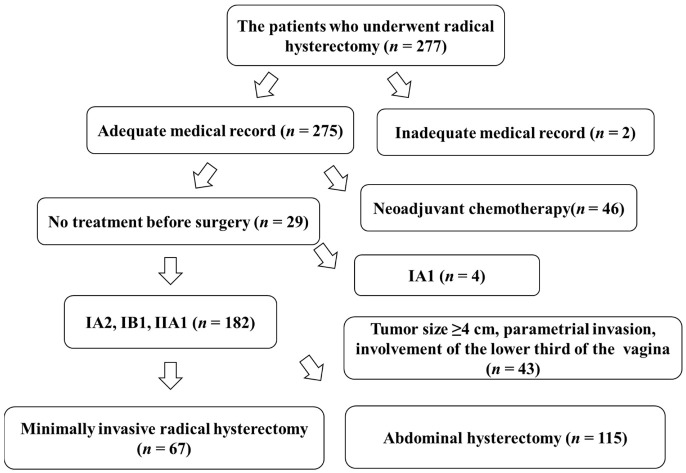
Chart of study participants.

There were 277 patients with cervical cancer who underwent either laparoscopic, robotic, or abdominal radical hysterectomy at the Educational Foundation of Osaka Medical and Pharmaceutical University from January 2013 to January 2021. Two hundred and seventy-five patients had complete information on their outcomes in their medical records. However, accurate medical records were not available for two patients, who were consequently excluded from the study. Forty-six patients underwent chemotherapy before surgery. Four patients had IA1 disease. Forty-three patients had disease with a tumor size ≥40 mm, involvement of the parametrium, or involvement of the lower third of the vagina; therefore, these patients were excluded from the study. A total of 182 patients had a stage of either IA2, IB1, or IIA1. Of these patients, 67 underwent MIS and 115 underwent ARH (Figure 3).

**Figure 4:**
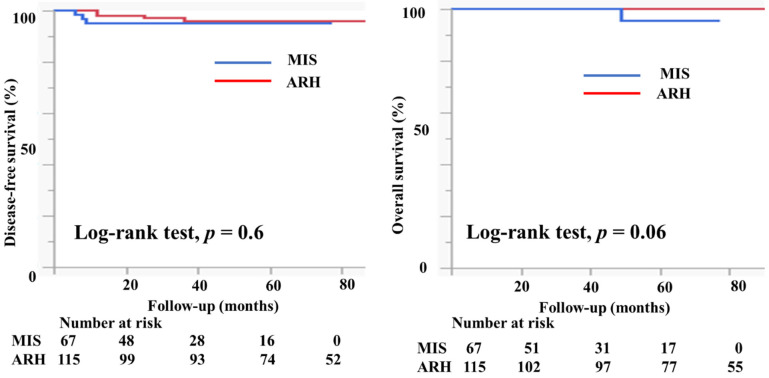
Prognosis of cervical cancer patients who underwent minimally invasive surgery and abdominal radical hysterectomy. The DFS and OS were not different between the groups (3y-DFS, 95.3% vs. 96.1%, *p* = 0.6; 3y-OS, 100% vs. 100%, *p* = 0.06) with the median follow-up of 33 (16–50) months for the MIS group and 80 (51–108) months for the ARH group.

Table 1 presents the characteristics of the study participants. Among 182 patients who met the study criteria, 67 underwent MIS (61 underwent laparoscopic surgery and six underwent robotic radical hysterectomy) and 115 patients underwent ARH. The mean age (44.6 ± 10.2 vs. 46.3 ± 10.4 years, *p* = 0.3) and the body mass index (21.9 ± 3.6 vs. 22.7 ± 3.9, *p* = 0.3) were not markedly different between the groups. In the MIS group, 11 (16.4%) patients had IA2 disease, 54 (80.6%) had IB1 disease, and two (3%) had IIA1 disease. In the ARH group, five (4.3%) patients had IA2 disease, 86 (74.8%) had IB1 disease, and 24 (20.9%) had IIA1 disease. Histologically, in the MIS group, 32 patients (47.8%) had squamous cell carcinoma, 33 (49.3%) had adenocarcinoma, and 2 (3%) had neuroendocrine carcinoma. Seventy-four patients in the ARH group (64.4%) had squamous cell carcinoma, 37 (32.2%) had adenocarcinoma, 2 (1.8%) had neuroendocrine carcinoma, and 2 (1.8%) had serous carcinoma; the rate of adenocarcinoma was higher in the MIS group than in the ARH group (49.3% vs. 32.2%). The mean tumor size (mm) was smaller in MIS patients than in ARH patients (15.3 ± 7.3 vs. 20.9 ± 9.5, *p* < 0.0001). The rate of lymph node metastasis did not differ between the groups (12.1% vs. 12.2%, *p* = 0.7). The rate of deep stromal invasion was lower in the MIS patients than in the ARH patients (18.2% vs. 41.7%, *p* = 0.001). The rate of lymph vascular involvement did not differ significantly between the groups (16.7% vs. 27.0%, *p* = 0.2). One patient in the MIS group had a positive cut end. The percentage of patients who underwent conization before radical hysterectomy was significantly higher in the MIS group than in the ARH group (52.2% vs. 25.2%, *p* = 0.0001). In the MIS group, 31 (46.3%) patients underwent sentinel navigation surgery and 36 (53.7%) underwent pelvic lymph node dissection. In contrast, all patients in the ARH group underwent pelvic lymph node dissection. In the MIS group, 47 (70.1%) patients did not undergo adjuvant therapy. Six (9.0%) patients underwent either radiotherapy (RT) or concurrent chemoradiotherapy (CCRT), and 14 (23.9%) patients underwent chemotherapy as an adjuvant therapy. In the ARH group, 48 (41.7%) patients did not undergo adjuvant therapy. Twenty-one (18.3%) patients underwent either radiotherapy or concurrent chemoradiotherapy, and 46 (40.0%) patients underwent chemotherapy as an adjuvant therapy. The median follow-up was 36 (18–60) months for patients in the MIS group and 78 (48–102) months for patients in the ARH group. The recurrence rate was 4.5% for patients in the MIS group and 3.5% for patients in the ARH group (Table 1). The DFS and OS were not significantly different between the groups (3y-DFS, 95.3% vs. 96.1%, *p* = 0.6; 3y-OS, 100% vs. 100%, *p* = 0.06, Figure 4).

**Figure 5:**
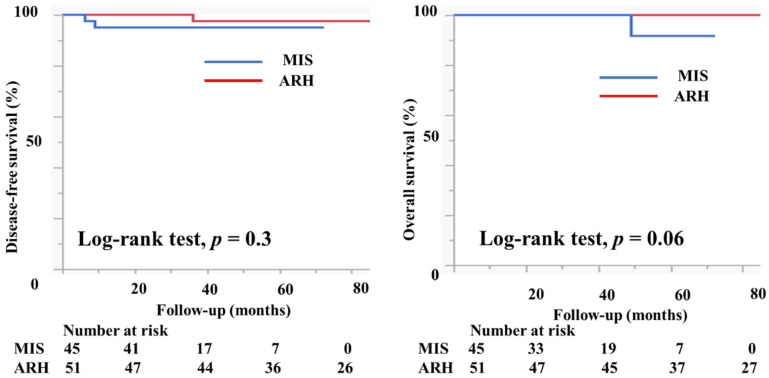
Prognosis of cervical cancer patients with tumors < 2 cm in size. The DFS and OS were not different between the groups (3y-DFS, 95.3% vs. 97.7%, *p* = 0.3; 3y-OS, 100% vs. 100%, *p* = 0.06) with the median follow-up of 33 (16–50) months for the MIS group and 80 (51–108) months for the ARH group.

Table 2 shows the characteristics of the patients who had tumors with a diameter of <2 cm. There were 45 patients in the MIS group and 51 patients in the ARH group. The mean tumor size (mm) was not different between the groups (11.2 ± 4.6 vs. 11.7 ± 4.1 mm, *p* = 0.6). The rates of lymph node metastasis (6.8% vs. 9.8%, *p* = 0.6), deep stromal invasion (6.7% vs. 13.7%, *p* = 0.2), lymph vascular involvement (13.3% vs. 9.8%, *p* = 0.6), and positive cut end (2.2% vs. 0%, *p* = 0.2) were not different between the groups. Among the MIS group, 37 (82.2%) patients did not undergo adjuvant therapy. Two (4.4%) patients underwent either RT or CCRT, and six (13.3%) patients underwent chemotherapy as an adjuvant therapy. Among the ARH group, 36 (70.6%) patients did not undergo adjuvant therapy. Two (3.9%) patients underwent either RT or CCRT, and 13 (25.5%) patients underwent chemotherapy as an adjuvant therapy. The median follow-up was 33 (16–50) months for patients in the MIS group and 80 (51–108) months for patients in the ARH group. The recurrence rate was 4.4% among patients in the MIS group and 2.0% among patients in the ARH group. The DFS and OS were not different between the groups (3y-DFS, 95.3% vs. 97.7%, *p* = 0.3; 3y-OS, 100% vs. 100%, *p* = 0.06, Figure 5). In the subgroup of patients with tumor size < 2 cm, the prognosis was not different between patients in the MIS and ARH groups.

**Figure 6:**
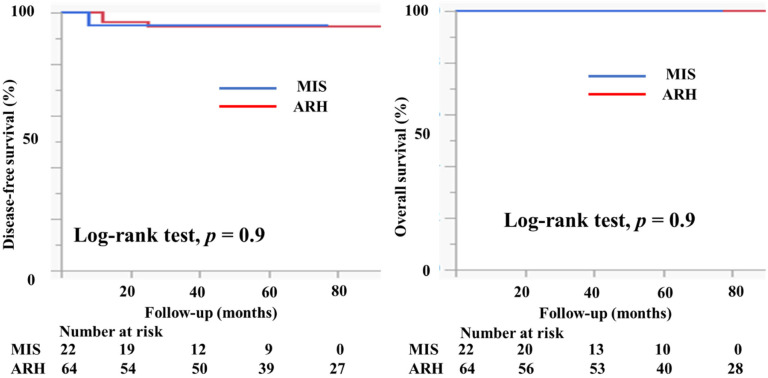
Prognosis of cervical cancer patients with tumors 2–4 cm in size. The DFS and OS were not different between the groups (3y-PFS, 95.2% vs. 94.8%, *p* = 0.9; 3y-OS, 100% vs. 100%) with the median follow-up of 51 (28–65) months for the MIS group and 75 (46–96) months for the ARH group.

Table 3 shows the characteristics of the patients who had tumors with a diameter of ≥2 cm. There were 22 patients in the MIS group and 64 patients in the ARH group. The mean tumor size (mm) was smaller in patients in the MIS group than in patients in the ARH group (23.8 ± 3.6 vs. 28.2 ± 5.1 mm, *p* = 0.0003). The rates of lymph node metastasis (22.7% vs. 14.1%, *p* = 0.4), deep stromal invasion (40.9% vs. 64.1%, *p* = 0.06), and lymph vascular involvement (22.7% vs. 40.6%, *p* = 0.1) were not different between the groups. No patient had a positive cut end in either group. In the MIS group, 10 (45.5%) patients did not undergo adjuvant therapy. Four (18.2%) patients underwent either RT or CCRT and eight (36.4%) underwent chemotherapy as an adjuvant therapy. In the ARH group, 11 (17.2%) patients did not undergo adjuvant therapy. Nineteen (29.7%) patients underwent either RT or CCRT and 34 (53.1%) underwent chemotherapy as an adjuvant therapy. The median follow-up was 51 (range, 28–65) months for patients in the MIS group and 75 (46–96) months for patients in the ARH group. The recurrence rate was 2.2% among patients in the MIS group and 4.7% among patients in the ARH group. The DFS and OS were not significantly different between the groups (3y-DFS, 95.2% vs. 94.8%, *p* = 0.9; 3y-OS, 100% vs. 100%, Figure 6).

**Figure 7:**
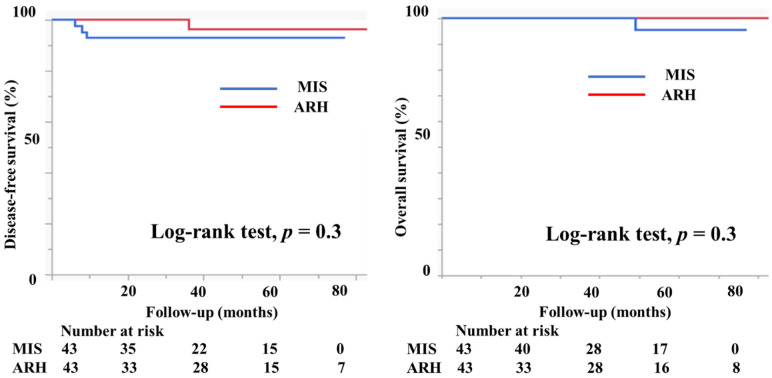
Prognosis of cervical cancer patients after propensity score-matching analysis. The DFS and OS were not different between the groups (3y-DFS, 93.0% vs. 96.4%, *p* = 0.3; 3y-OS, 100% vs. 100%, *p* = 0.3).

A propensity score-matching analysis was performed using several parameters, including vaginal invasion, histological type, tumor size, lymph node metastasis, deep stromal invasion, lymph vascular involvement, conization, and adjuvant therapy. After the analysis, 43 patients in the MIS group and 43 in the ARH group were selected for DFS comparison. Similarly, 43 patients with MIS and 43 patients with ARH were selected for OS comparison (Table 4). Figure 7 displays the prognosis of both groups after the propensity score analysis. DFS and OS were not significantly different between the groups (3y-DFS, 93.0% vs. 96.4%, *p* = 0.3; 3y-OS, 100% vs. 100%, *p* = 0.3, Figure 7).

## 4. Discussion

In the current study, MIS with vaginal closure did not lead to a poorer prognosis than ARH. In subgroups according to tumor size, prognosis did not differ between the groups. Surgical techniques and procedures that avoid spillage of tumor cells could be important for a better prognosis.

The LACC trial, which is the largest prospective study to compare MIS with ARH, showed that MIS resulted in a poorer prognosis compared to ARH [2]. The results of that study surprised the gynecologic oncologist because they believed that MIS was not inferior to ARH. The use of MIS has been increasing in the last decade because of its minimally invasive nature [1]. The NCCN guidelines recommend that patients with cervical cancer be carefully counseled about the oncologic risk and potential short-term benefits of the different surgical approaches; this means that ARH is the standard for patients with cervical cancer [6]. The LACC trial is a prospective randomized control trial involving 319 MIS patients, 16% of which underwent robotic surgery, and 312 ARH cervical cancer patients. Ninety-two percent of patients in both groups had stage IB1 disease. MIS patients had poorer DFS (3y-DFS, 91.2% vs. 97.1%; hazard ratio (HR) for recurrence, 3.74; 95% confidence interval (CI), 1.63–8.58) and OS (3-y OS, 93.8% vs. 99.0%; HR for death, 6.00; 95% CI, 1.77–20.3) [2].

Based on the findings of the LAAC trial, several epidemiologic cohort studies were performed. These studies used either the National Cancer Database (NCDB), which contains data from patients who were treated at the Commission on Cancer-accredited centers and contains approximately 70% of newly diagnosed cancer cases in more than 1500 hospitals in the United States, or the Surveillance, Epidemiology, and End Results (SEER), which is a population-based cancer registry that covers 28% of the United States population [1]. Melamed et al. analyzed survival after MIS for early cervical cancer using the NCDB and SEER. The study included IA2 and IB1 cervical cancer patients who underwent MIS and ARH. A total of 1225 patients underwent MIS and 1236 patients underwent ARH. The data were adjusted. Afterwards, approximately 41% of patients had tumors < 2 cm in size and 47% had tumors ≥ 2 cm in size in both groups. Over a median follow-up of 45 months, the four-year mortality was 9.1% among women who underwent MIS and 5.3% among those who underwent ARH (HR, 1.65; 95% CI, 1.22–2.22). The rate of MIS was less than 5% in 2006, increasing to 30% in 2010. The adoption of MIS coincided with the beginning of a decline in the four-year relative survival rate of 0.8% per year from 2006 to 2010 [1]. Margul et al. analyzed the oncologic outcomes of cervical cancer patients at stage IB1 from 2010 to 2013 based on the NCDB. There were 910 patients who underwent MIS and 982 patients who underwent ARH. Although MIS was associated with decreased surgical morbidity and costs, patients with tumor size ≥ 2 cm who underwent MIS had decreased five-year survival compared to those who underwent open radical hysterectomy (81.3% vs. 90.8%, *p* < 0.001) [7].

Recent studies [8,9], including meta-analyses [10,11,12] and systematic reviews [11,12], demonstrate that MIS is associated with poor survival outcomes compared to open surgery. However, several authors found that tumor size is an important prognostic factor for cervical cancer patients who underwent MIS; patients with tumor sizes < 2 cm might benefit from MIS [10,13,14]. Furthermore, several authors suggested that surgeon experience [14], preoperative conization [14] and intrauterine manipulator use [8] are also important prognostic factors. For example, Kohler et al. found that in 389 patients with early cervical cancer treated with laparoscopic-assisted radical vaginal hysterectomy or vaginal-assisted laparoscopic radical hysterectomy, with strict uterine manipulator avoidance and use of vaginal cuff covering the rumor, the oncologic outcomes were nearly identical to the excellent results in the LACC trial [15]. In contrast, Chiva et al. evaluated 1272 patients who underwent radical hysterectomy surgery by an open or a minimally invasive approach for stage IB1 cervical cancer. Patients who underwent minimally invasive surgery using a uterine manipulator had a 2.76-times higher risk of relapse (HR, 2.76; 95% CI, 1.75 to 4.33; *p* <0.001), and those without the use of a uterine manipulator had similar disease-free -survival to the open surgery group (HR, 1.58; 95% CI, 0.79 to 3.15; *p* = 0.20) [8]. The hypothesis of vaginal closure or uterine manipulator avoidance as an oncological hygiene strategy is promising and should be validated in prospective studies. These data suggest that surgeon experience, small tumor size, and surgical technique to avoid tumor spillage are important in order to improve prognosis. We previously reported that there was no tumor spillage in cervical cancer patients who underwent laparoscopic radical hysterectomy with vaginal closure [3]. Some authors also reported that the use of a technique that helps to avoid tumor spillage is important [8,16]. In the current study, only two surgeons who were specialists in gynecologic surgery performed the MIS with vaginal closure. Furthermore, the prognosis was not different between the groups in the subgroups according to size of tumor; the oncologic outcomes of the patients who underwent MIS with vaginal closure in our institution were excellent.

The present study has several limitations that cannot be overlooked. First, the sample size was relatively small. Second, the evaluation of lymph node status was not the same between the groups. Third, there was a bias when assigning patients to either the MIS or ARH group. Fourth, although the currently recommended adjuvant treatment is radiotherapy or chemoradiotherapy, many patients receive chemotherapy as an adjuvant therapy. Fifth, the global follow-up and tumor size between were very different between the groups. Given that this was a retrospective observational comparative study, our results must be confirmed through further investigation, such as through prospective cohort studies. 

## 5. Conclusions

MIS with vaginal closure did not show a poorer prognosis than ARH. In the subgroups according to tumor size, the prognosis was not different between the groups. Surgical techniques and procedures to avoid spillage of tumor cells could be important for a better prognosis.

## Figures and Tables

**Table 1:** Characteristics of the patients who underwent radical hysterectomy.

	MIS (%)	ARH (%)	*p* Value
Number of patients	67	115	
Age, years old *	44.6 ± 10.2	46.3 ± 10.4	0.3
BMI *	21.9 ± 3.6	22.7 ± 3.9	0.3
FIGO stage			
IA2	11 (16.4)	5 (4.3)	
IB1	54 (80.6)	86 (74.8)	
IIA1	2 (3.0)	24 (20.9)	0.006
Histological type			
Squamous cell carcinoma	32 (47.8)	74 (64.4)	
Adenocarcinoma	33 (49.3)	37 (32.2)	
Others	2 (3.0)	4 (3.5)	0.2
Tumor size, mm *	15.3 ± 7.3	20.9 ± 9.5	<0.0001
Lymph node metastasis	8 (12.1)	14 (12.2)	0.7
Deep stromal invasion	12 (18.2)	48 (41.7)	0.001
Lymph vascular involvement	11 (16.7)	32 (27.0)	0.2
Positive cut end	1 (1.5)	0	0.4
Conization	35 (52.2)	29 (25.2)	0.001
Lymph nodes assessment			
PLD	36 (53.7)	115 (100)	
SNNS	31 (46.3)	0	<0.0001
No adjuvant therapy	47 (70.1)	48 (41.7)	
Adjuvant RT or CCRT	6 (9.0)	21 (18.3)	
Adjuvant chemotherapy	14 (23.9)	46 (40.0)	0.003
Follow-up, median months (IQR)	36 (18–60)	78 (48–102)	<0.001
Recurrence (%)	3 (4.5)	4 (3.5)	0.9
3-year DFS	95.3	96.1	0.6
3-year OS	100	100	0.06

* According to an analysis of variance (mean ± standard deviation); MIS, minimally invasive surgery; ARH, abdominal radical hysterectomy; BMI, body mass index; FIGO, the Federation of Gynecology and Obstetrics; PLD, pelvic lymph node dissection; SNNS, sentinel node navigation surgery; RT, radiotherapy; CCRT, concurrent chemoradiotherapy; IQR, interquartile range; DFS, disease-free survival; OS, overall survival.

**Table 2:** Characteristics of the patients with tumors < 2 cm in size.

	MIS (%)	ARH (%)	*p* Value
Number of patients	45	51	
Tumor size, mm *	11.2 ± 4.6	11.7 ± 4.1	0.6
Lymph node metastasis	3 (6.8)	5 (9.8)	0.6
Deep stromal invasion	3 (6.7)	7 (13.7)	0.2
Lymph vascular involvement	6 (13.3)	5 (9.8)	0.6
Positive cut end	1 (2.2)	0	0.2
No adjuvant therapy	37 (82.2)	36 (70.6)	
Adjuvant RT or CCRT	2 (4.4)	2 (3.9)	
Adjuvant chemotherapy	6 (13.3)	13 (25.5)	0.3
Follow-up, median months (IQR)	33 (16–50)	80 (51–108)	<0.001
Recurrence (%)	2 (4.4)	1 (2.0)	0.5
3-year DFS	95.3	97.7	0.3
3-year OS	100	100	0.06

* According to an analysis of variance (mean ± standard deviation); MIS, minimally invasive surgery; ARH, abdominal radical hysterectomy; RT, radiotherapy; CCRT, concurrent chemoradiotherapy; IQR, interquartile range; DFS, disease-free survival; OS, overall survival.

**Table 3:** Characteristics of the patients with tumors 2–4 cm in size.

	MIS (%)	ARH (%)	*p* Value
Number of patients	22	64	
Tumor size, mm *	23.8 ± 3.7	28.2 ± 5.1	0.0003
Lymph node metastasis	5 (22.7)	9 (14.1)	0.4
Deep stromal invasion	9 (40.9)	41 (64.1)	0.06
Lymph vascular involvement	5 (22.7)	26 (40.6)	0.1
Positive cut end	0	0	
No adjuvant therapy	10 (45.5)	11 (17.2)	
Adjuvant RT or CCRT	4 (18.2)	19 (29.7)	
Adjuvant chemotherapy	8 (36.4)	34 (53.1)	0.04
Follow-up, median months (IQR)	51 (28–65)	75 (46–96)	0.004
Recurrence (%)	1 (2.2)	3 (4.7)	0.9
3-year DFS	95.2	94.8	0.9
3-year OS	100	100	

* According to an analysis of variance (mean ± standard deviation); MIS, minimally invasive surgery; ARH, abdominal radical hysterectomy; RT, radiotherapy; CCRT, concurrent chemoradiotherapy; IQR, interquartile range; DFS, disease-free survival; OS, overall survival.

**Table 4:** Characteristics of the patients after propensity score-matching analysis.

	DFS			OS		
	MIS (%)	ARH (%)	*p* Value	MIS (%)	ARH (%)	*p* Value
Number of patients	43	43		43	43	
Histological type						
Squamous cell carcinoma	27 (62.8)	21 (48.8)		24 (55.8)	21 (48.8)	
Adenocarcinoma	16 (37.2)	22 (51.2)	0.2	19 (44.2)	22 (51.2)	0.5
Vaginal invasion	2 (4.7)	3 (7.0)	0.6	2 (4.7)	3 (7.0)	0.6
Lymph node metastasis	6 (14.0)	5 (11.6)	0.7	6 (14.0)	5 (11.6)	0.7
Deep stromal invasion	10 (23.3)	12 (27.9)	0.6	12 (27.9)	12 (27.9)	1.0
Lymph vascular involvement	7 (16.3)	8 (18.6)	0.8	9 (20.9)	8 (18.6)	0.8
Tumor size, mm *	16.5 ± 7.9	18.0 ± 9.4	0.4	16.8 ± 7.5	18.0 ± 9.4	0.5
Conization	18 (41.9)	13 (30.2)	0.3	16 (37.2)	13 (30.2)	0.5
Adjuvant therapy	15 (34.9)	19 (44.2)	0.4	15 (34.9)	19 (44.2)	0.4
Follow-up, median months (IQR)	48 (27–64)	49 (19–74)	0.5	49 (28–65)	49 (19–74)	0.7

* According to an analysis of variance (mean ± standard deviation); MIS, minimally invasive surgery; ARH, abdominal radical hysterectomy; IQR, interquartile range.

## Data Availability

The data presented in this study are available on request from the corresponding author.
